# Deep learning models for automatic tumor segmentation and total tumor volume assessment in patients with colorectal liver metastases

**DOI:** 10.1186/s41747-023-00383-4

**Published:** 2023-12-01

**Authors:** Nina J. Wesdorp, J. Michiel Zeeuw, Sam C. J. Postma, Joran Roor, Jan Hein T. M. van Waesberghe, Janneke E. van den Bergh, Irene M. Nota, Shira Moos, Ruby Kemna, Fijoy Vadakkumpadan, Courtney Ambrozic, Susan van Dieren, Martinus J. van Amerongen, Thiery Chapelle, Marc R. W. Engelbrecht, Michael F. Gerhards, Dirk Grunhagen, Thomas M. van Gulik, John J. Hermans, Koert P. de Jong, Joost M. Klaase, Mike S. L. Liem, Krijn P. van Lienden, I. Quintus Molenaar, Gijs A. Patijn, Arjen M. Rijken, Theo M. Ruers, Cornelis Verhoef, Johannes H. W. de Wilt, Henk A. Marquering, Jaap Stoker, Rutger-Jan Swijnenburg, Cornelis J. A. Punt, Joost Huiskens, Geert Kazemier

**Affiliations:** 1 Department of Surgery, Cancer Center Amsterdam, Amsterdam UMC, Vrije Universiteit Amsterdam, De Boelelaan 1117, 1081 HV Amsterdam, the Netherlands; 2 Department of Health, SAS Institute B.V, Huizen, the Netherlands; 3 Department of Radiology and Nuclear Medicine, Cancer Center Amsterdam, Amsterdam UMC, Vrije Universiteit Amsterdam, Amsterdam, the Netherlands; 4 Department of Computer Vision and Machine Learning, SAS Institute Inc, Cary, NC USA; 5 Department of Radiology, Sint Maartenskliniek, Nijmegen, the Netherlands; 6 Department of Hepatobiliary, Transplantation, and Endocrine Surgery, Antwerp University Hospital, Antwerp, Belgium; 7 Department of Surgery, OLVG Hospital, Amsterdam, the Netherlands; 8 Department of Surgical Oncology and Gastrointestinal Surgery, Erasmus MC Cancer Institute, Rotterdam, the Netherlands; 9 Department of Medical Imaging, Radboud University Medical Center, Radboud University Nijmegen, Nijmegen, the Netherlands; 10 Department of HPB Surgery and Liver Transplantation, University of Groningen, University Medical Center Groningen, Groningen, the Netherlands; 11 Department of Surgery, Medical Spectrum Twente, Enschede, the Netherlands; 12 Department of Interventional Radiology, St Antonius Hospital, Nieuwegein, the Netherlands; 13 Department of Surgery, Regional Academic Cancer Center Utrecht, University Medical Center Utrecht, Utrecht, the Netherlands; 14 Department of Surgery, St Antonius Hospital, Nieuwegein, the Netherlands; 15 Department of Surgery, Isala Hospital, Zwolle, the Netherlands; 16 Department of Surgery, Amphia Hospital, Breda, the Netherlands; 17 Department of Surgery, Radboud University Medical Center, Radboud University Nijmegen, Nijmegen, the Netherlands; 18 Department of Biomedical Engineering and Physics, Amsterdam UMC, University of Amsterdam, Amsterdam, the Netherlands; 19 Department of Medical Oncology, Cancer Center Amsterdam, Amsterdam UMC, University of Amsterdam, Amsterdam, the Netherlands; 20 Department of Epidemiology, Julius Center for Health Sciences and Primary Care, University Medical Center Utrecht, Utrecht, the Netherlands

**Keywords:** Artificial intelligence, Deep learning, Colorectal cancer, Liver neoplasms, Tomography (x-ray computed)

## Abstract

**Background:**

We developed models for tumor segmentation to automate the assessment of total tumor volume (TTV) in patients with colorectal liver metastases (CRLM).

**Methods:**

In this prospective cohort study, pre- and post-systemic treatment computed tomography (CT) scans of 259 patients with initially unresectable CRLM of the CAIRO5 trial (NCT02162563) were included. In total, 595 CT scans comprising 8,959 CRLM were divided into training (73%), validation (6.5%), and test sets (21%). Deep learning models were trained with ground truth segmentations of the liver and CRLM. TTV was calculated based on the CRLM segmentations. An external validation cohort was included, comprising 72 preoperative CT scans of patients with 112 resectable CRLM. Image segmentation evaluation metrics and intraclass correlation coefficient (ICC) were calculated.

**Results:**

In the test set (122 CT scans), the autosegmentation models showed a global Dice similarity coefficient (DSC) of 0.96 (liver) and 0.86 (CRLM). The corresponding median per-case DSC was 0.96 (interquartile range [IQR] 0.95–0.96) and 0.80 (IQR 0.67–0.87). For tumor segmentation, the intersection-over-union, precision, and recall were 0.75, 0.89, and 0.84, respectively. An excellent agreement was observed between the reference and automatically computed TTV for the test set (ICC 0.98) and external validation cohort (ICC 0.98). In the external validation, the global DSC was 0.82 and the median per-case DSC was 0.60 (IQR 0.29–0.76) for tumor segmentation.

**Conclusions:**

Deep learning autosegmentation models were able to segment the liver and CRLM automatically and accurately in patients with initially unresectable CRLM, enabling automatic TTV assessment in such patients.

**Relevance statement:**

Automatic segmentation enables the assessment of total tumor volume in patients with colorectal liver metastases, with a high potential of decreasing radiologist’s workload and increasing accuracy and consistency.

**Key points:**

• Tumor response evaluation is time-consuming, manually performed, and ignores total tumor volume.

• Automatic models can accurately segment tumors in patients with colorectal liver metastases.

• Total tumor volume can be accurately calculated based on automatic segmentations.

**Graphical Abstract:**

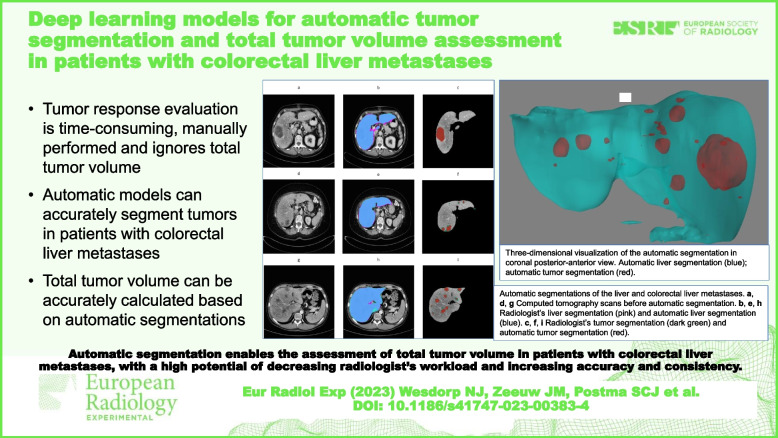

**Supplementary Information:**

The online version contains supplementary material available at 10.1186/s41747-023-00383-4.

## Background

Response to systemic treatment of solid tumors is currently assessed using the Response Evaluation Criteria in Solid Tumors (RECIST1.1) [1, 2]. According to RECIST1.1, response to treatment is measured as the change in the sum of diameters in two target lesions per organ. RECIST1.1 aims to perform an objective assessment of tumor change, but the measurements are performed manually. This is not only tedious and time-consuming, but also subjective. The subjective nature of RECIST 1.1 leads to nonnegligible inter- and intra-observer variability [3, 4].

In patients with colorectal liver metastases (CRLM), the efficacy of RECIST1.1 has been questioned [5–7]. Colorectal cancer is the third most common cancer and the second leading cause of cancer-related deaths for men and women globally [8]. Almost half of these patients develop CRLM during the course of their illness [9–11]. For patients with CRLM, treatment response evaluation is crucial, as approximately 80% of these patients are not suitable for a potential curative local treatment at diagnosis [12, 13]. Patients with unresectable CRLM most often receive systemic treatment in a palliative setting or in a neoadjuvant setting to induce downsizing of the tumor load. Patients with initially unresectable liver-only CRLM can become eligible for local treatment with curative intent by systemic induction treatment in approximately 25% of cases [14–16].

Treatment decision-making for patients with CRLM is predominantly based on arguments involving technical resectability [17]. The question remains if local treatment such as surgery is clinically relevant for the individual patient. There is a growing interest in how a shift can be made from technically driven surgery to biologically driven surgery. Biologically driven surgery aims to select patients for the most optimal treatment to achieve long-term survival, taking into consideration tumor biology [18]. By doing so, the effects of systemic therapy could be underestimated by RECIST1.1, as it ignores potentially valuable information about total tumor volume (TTV). Assessment of TTV response to systemic therapy could represent a clinically more reliable evaluation since baseline TTV has shown to be prognostic for overall survival and change in TTV for recurrence-free survival in patients with CRLM, whereas RECIST1.1 has not [6, 7, 19].

In recent years, several studies demonstrated that volumetric assessment using algorithms increases the reproducibility of response assessments [6, 20–22]. In most of these studies, semiautomatic segmentation models are used to perform volumetric assessments [6, 7, 20–22]. Segmentation is the delineation of tissue structures on diagnostic imaging, resulting in 3D contours of these structures. The use of semiautomatic models, however, is still time-consuming and would be too labor-intensive to perform in daily practice. Fully automatic segmentation models could enable the automation of TTV evaluation.

Numerous autosegmentation models have been developed for the segmentation of livers and liver tumors on computed tomography (CT) or magnetic resonance imaging (MRI) [23]. Most studies on autosegmentation of liver tumors used imaging data from the Liver Tumor Segmentation Challenge (LiTS) [24–27]. The LiTS was conducted to compare state-of-the-art automated liver and tumor segmentation methods, and the dataset contained imaging data of various types of liver tumors [25]. For response monitoring of CRLM, it is far more important to optimize the performance for this disease, than for a wide range of tumors. Focusing on autosegmentation of CRLM, Vorontsov et al. developed a deep learning model with variable performance with Dice similarity coefficient (DSC) ranging from 0.14 to 0.68, depending on lesion size [27]. This model was trained and validated on CT scans of various liver tumors and tested on a small dataset of 26 CT scans comprising patients with CRLM. We hypothesize that with a larger and homogeneous population of patients suffering from CRLM only, the performance of deep learning-based tumor and liver segmentation can be improved.

In this study, we aim to develop deep learning models for automatic tumor segmentation of CRLM and the liver using a comprehensive training and test set of patients with initially unresectable CRLM. The secondary aim is to automate the assessment of TTV response to systemic therapy in such patients.

## Methods

### Development cohort

#### Study population

In this prospective cohort study, patients registered between November 2014 and April 2019 from the ongoing multicenter randomized clinical trial of the Dutch Colorectal Cancer Group, CAIRO5 (NCT02162563), were included for model development and testing [28]. The CAIRO5 trial aims to select the optimal systemic induction therapy for patients with initially unresectable liver-only CRLM (Additional file 1: S1). Patients are randomized between different systemic therapy combinations based on primary tumor site and genetic mutation status (*RAS/BRAF*). Treatment regimens consist of doublet or triplet chemotherapy in combination with targeted therapy. All included patients signed a written informed consent form, also allowing side studies such as the current one.

#### Imaging

**Fig. 1:**
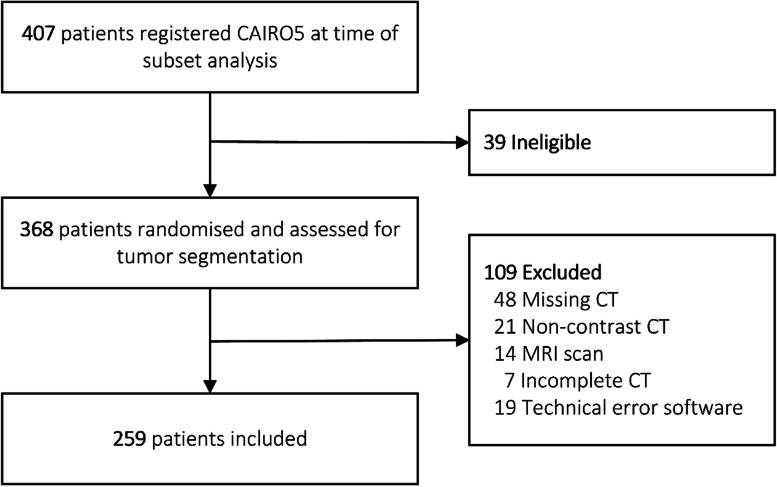
Patient selection of development cohort. *CT* Computed tomography, *MRI* Magnetic resonance imaging. *The patients excluded because of “MRI scan” had a MRI scan instead of a CT scan for their diagnostic work-up. For patients with “Missing CT,” the baseline or follow-up CT scan was not available. The error in segmentation software occurred in the IntelliSpace Portal software of Philips

Imaging data of this development cohort consisted of contrast-enhanced CT scans of the chest and abdomen at baseline and every 2 months during systemic therapy. All scans were performed in one of the 54 medical centers responsible for inclusion using different types of CT scanners and acquisition protocols. In the current study, only patients with contrast-enhanced abdominal CT scans in the portal venous phase were included (Fig. 1). Exclusion criteria were non-contrast enhanced or not portal venous CT scan, missing or incomplete CT scan, the use of MRI or ^18^F-fluorodeoxyglucose positron emission tomography instead of portal venous CT, and technical error in segmentation software. CT acquisition characteristics are summarized in Additional file 1: S2.

### Data processing

#### Reference segmentations

All available pre- and post-treatment CT scans of the development cohort were used for semi-automatic segmentation of the liver and CRLM in the Tumor Tracking Modality of IntelliSpace Portal 9.0® (Philips Healthcare, Best, the Netherlands). In all CT scans, the liver and all CRLM were segmented by one of three trained members of the research team (N.J.W., S.P., R.K.). Lesions were roughly outlined, which resulted in a semi-automatic contour or region of interest based on differences in density. These contours were subsequently manually adjusted in every slice for accurate segmentation. All segmentations performed by the trained research team were verified and, if needed, adjusted by an abdominal radiologist with 18 years of experience (J.H.T.M.W.). Three abdominal radiologists with 10 (J.E.B.), 2 (I.M.N.), and 1 (S.I.M.) years of experience also independently corrected and verified 41 scans of 20 patients segmented by a member of the research team.

#### Image processing steps

The DICOM files of the CT scans and the DICOM-RT files of the 3D semi-automatic segmentations were uploaded into the SAS Viya® Analytical Platform (SAS Viya 3.5, SAS Institute Inc.). The scans and segmentations were combined to create liver and tumor masks which were used as target segmentation maps. The density values were adjusted by clipping and histogram equalization. Firstly, clipping between -100 and 400 Hounsfield units was performed to restrict the density values to a common range in the liver. Secondly, histogram equalization was applied to better distribute the image histogram, utilizing the full range of Hounsfield units in the histogram for every image evenly.

### Development and testing of autosegmentation models

**Fig. 2:**
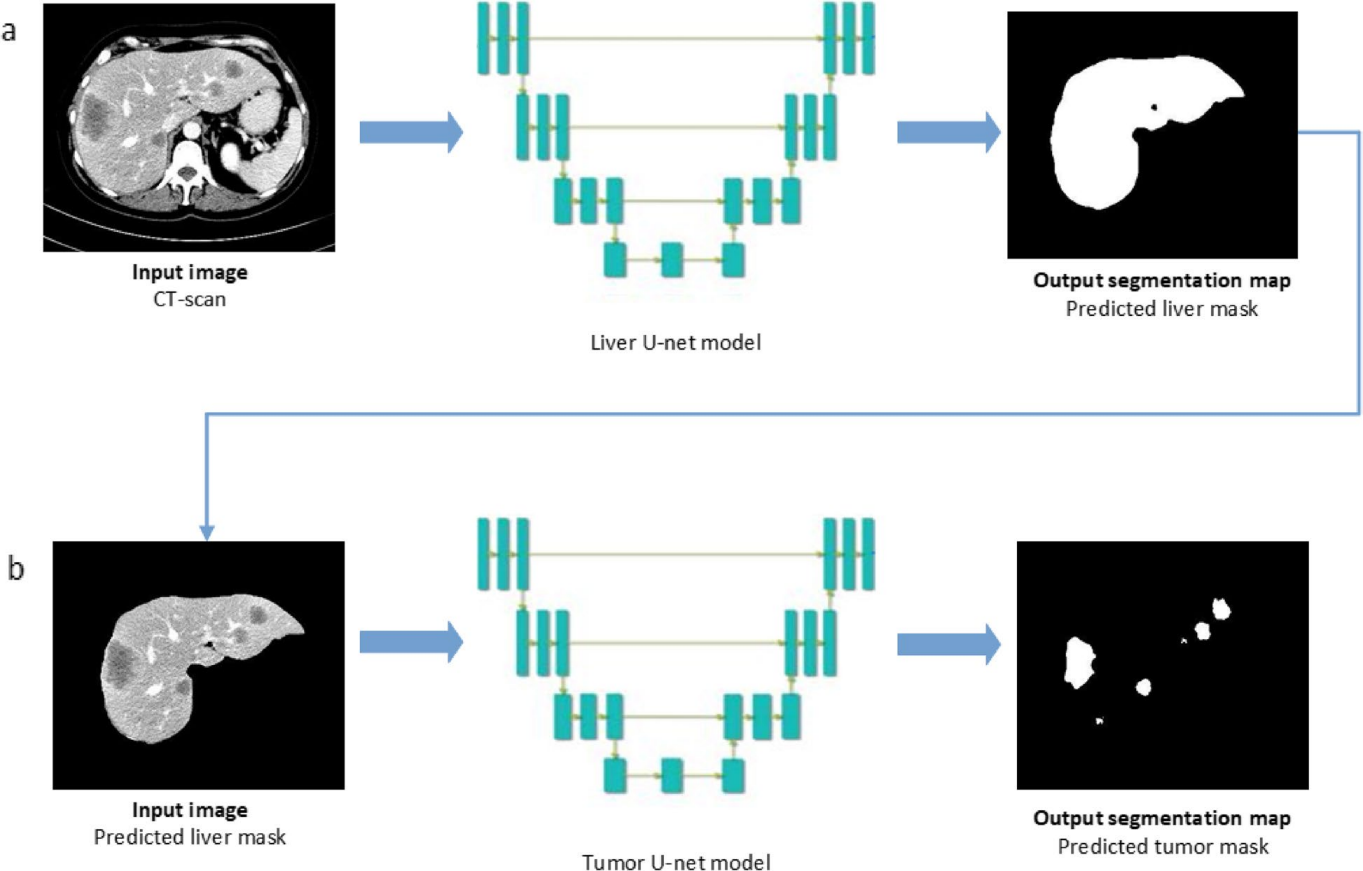
Automatic segmentation process. **a** The liver U-net model receives the computed tomography scan as input image. The output of the liver U-net model is the automatic liver segmentation. **b** The automatic liver segmentation is used as the volume of interest for the tumor U-net model. The output of the tumor U-net model is the automatic tumor segmentation

The U-net architecture was used for the segmentation models (Additional file 1: S3). Two U-nets were trained and tested, one for liver and one for tumor segmentation. Liver segmentation was performed to restrict the volume of interest for tumor segmentation. Model training, validation, and testing were performed within the SAS Viya® Analytical Platform. The radiologist’s segmentations of the liver and CRLM from the development cohort were used as reference data. A total of 434 (72.8%) CT scans with 6,667 CRLM were randomly assigned to the training set, 39 (6.5%) CT scans with 487 CRLM in the validation set, and 122 (20.6%) CT scans with 1,805 CRLM in the test set (Additional file 1: S4). The validation set was used for performance evaluation during training and to determine stop criteria. It was ensured that no image data of the same patient was included in both the training/validation set and the test set. This was done to prevent data leakage between the training/validation set and the test set. The automated liver segmentations were used as the volumes of interest for the autosegmentation tumor model (Fig. 2).

### External validation

The tumor segmentation model performance was assessed with an external population of patients with CRLM. The CRLM dataset of the publicly available Workflow for Optimal Radiomics Classification, WORC, was used. This dataset consists of preoperative CT scans in the portal venous phase of 77 patients, surgically treated at the Erasmus University MC Rotterdam, the Netherlands (Additional file 1: S5) [29, 30]. All CRLM in the CT scans were segmented by one of the members of the research team and verified and if needed adjusted by an abdominal radiologist (J.H.T.M.W.) using IntelliSpace Portal 9.0® [31]. In addition, all livers and CRLM were automatically segmented by the developed models in the SAS Viya® Analytical Platform [32].

### Statistics

The performances of the autosegmentation models and the segmentation agreement between different observers were assessed using the Dice similarity coefficient (DSC) as an accuracy measure, ranging between 0 (no overlap) and 1 (complete overlap) [33]. Two DSCs were calculated: the global DSC, which is the DSC of all CT scans combined, and the per-case DSC, which is the average per-CT scan DSC. Intersection-over-union, precision, and recall were also calculated. The summary statistics were calculated with formulas proposed by LiTS [25]. Total tumor volume was calculated in the SAS Viya® Analytical platform using the *quantifyBioMedImages* action [7, 34]. Total tumor volume was determined as the product of the voxel volume and the number of segmented voxels of all CRLM present in the liver and was reported as a continuous variable in cubic centimeters. A two-way mixed effect intraclass correlation coefficient (ICC) for absolute agreement was calculated to compare the reference and automatically computed TTV. The ICC was categorized as having either poor (ICC < 0.40), fair (ICC 0.40–0.59), good (ICC 0.60–0.74), or excellent (ICC 0.75–1.0) agreement [35, 36]. The distribution of normality of continuous variables was checked by visually inspecting the histograms and boxplots. Continuous variables were reported as median with interquartile range (IQR) and compared with Mann–Whitney *U* or *t* test, as appropriate. Categorical variables were displayed as frequencies and percentages and compared with chi-square test or Fisher’s exact test, as appropriate. Test results were considered statistically significant with a *p* < 0.05. Statistical analyses were performed using SAS® Studio (version 5.2, SAS Viya® 03.05).

## Results

### Study population

In total, 259 of 407 patients from the CAIRO5 trial were included in the development cohort of this study. The most common reason for exclusion was a missing CT scan, and 39 patients were not eligible because of not meeting inclusion criteria or withdrawal from the study (Fig. 1). Of all 259 patients, a baseline and first follow-up CT scan were available for analysis. In some cases, two or three follow-up scans were available and included. In total, 595 CT scans were included and 8,959 CRLM were segmented. In the development cohort, the median age was 62 (IQR 55−71) years and 36% (94/259) of the patients were female. Per patient, the median number of CRLM at baseline was 11 (IQR 7−21), with a median of six liver segments involved (IQR 4−7). Significant differences between training/validation and test set were observed, as a larger number of males were allocated in the training cohort, and the largest diameter of CRLM was smaller in the test set (Table 1). In the external validation cohort, a total of 72 patients with 112 CRLM were included. Five patients were excluded (Additional file 1: S5). The median age was 68 (IQR 59−77) years, 42% (30/72) of the patients were female, and the median number CRLM was 1 (IQR 1−2).

**Table 1:** Baseline patient and tumor characteristics of development CAIRO5 cohort

Baseline parameters	Total cohort	Training cohort	Test set	*p* value
	*n* = 259	*n* = 206	*n* = 53	
Age (years)	62 [55–71]	62 [55–71]	63 [56–71]	0.956
Sex				
Male	165 (63.7)	123 (59.7)	42 (79.2)	0.008
Female	94 (36.3)	83 (40.3)	11 (20.8)	
Site of the primary tumor				
Right colon	74 (28.6)	61 (29.6)	13 (24.5)	0.465
Left colon or rectum	185 (71.4)	145 (70.4)	40 (75.7)	
Time to metastases				
Synchronous	228 (88.0)	182 (88.3)	46 (86.8)	0.755
Metachronous	31 (12.0)	24 (11.7)	7 (13.2)	
Mutational status				
*RAS/BRAF* mutation	154 (59.5)	125 (60.7)	29 (54.7)	0.430
*RAS/BRAF* wild-type	105 (40.5)	81 (39.3)	24 (45.3)	
Number of liver metastases	11 [7–21]	11 [7–23]	12 [7–20]	0.890
Diameter of largest metastasis (mm)	41 [28–72]	46 [29–73]	34 [26–50]	**0.011**
Number of liver segments	6 [4–7]	6 [4–7]	6 [5–7]	0.397
Distribution of liver metastases				
Unilobar	19 (7.3)	18 (8.7)	1 (1.9)	0.088
Bilobar	240 (92.7)	188 (91.3)	52 (98.1)	
Induction systemic therapy				
FOLFOX/FOLFIRI and Bevacizumab	129 (49.8)	105 (51.0)	24 (45.3)	0.751
FOLFOX/FOLFIRI and Panitumumab	51 (19.7)	40 (19.4)	11 (20.8)	
FOLFOXIRI and Bevacizumab	79 (30.5)	61 (29.6)	18 (34.0)	

Values are shown as median (interquartile range, 25th − 75th percentile) or number of participants (percentage). Training cohort consists of CT scans from the training set and validation set, as both sets were used for model training*. BRAF* v-Raf murine sarcoma viral oncogene homolog B, *FOLFIRI* 5-fluoracil with leucovorin and irinotecan, *FOLFOX* 5-fluoracil with leucovorin and oxaliplatin, *FOLFOXIRI* 5-fluoracil with leucovorin, oxaliplatin and irinotecan, *RAS* Rat sarcoma oncogene

### Accuracy of autosegmentation models

**Fig. 3:**
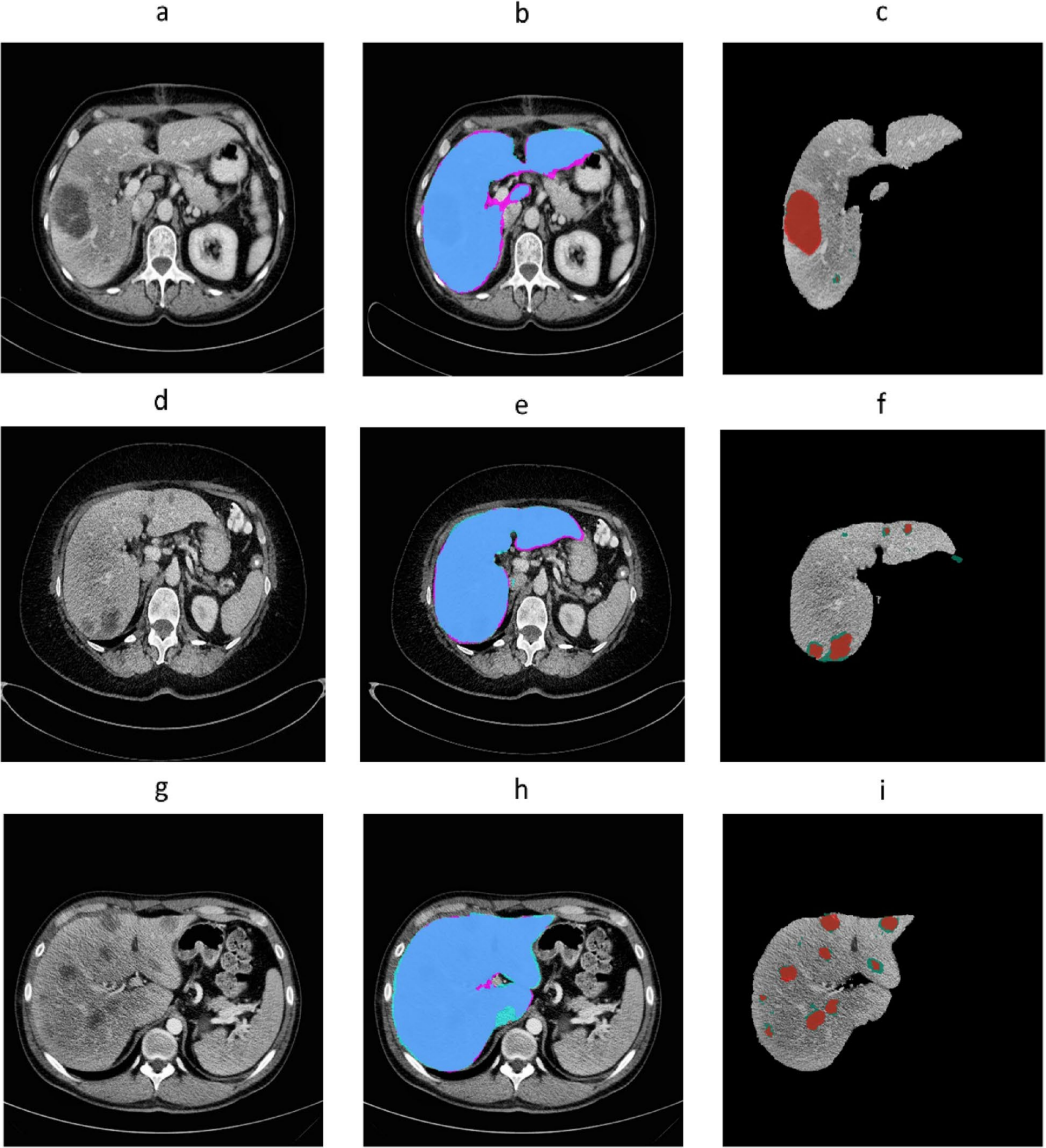
Automatic segmentations of the liver and colorectal liver metastases in three patients of the development cohort. **a**, **d**, **g** Computed tomography scans before automatic segmentation. **b**, **e**,** h** Radiologist’s liver segmentation (pink) and automatic liver segmentation (blue). **c**, **f**, **i** Radiologist’s tumor segmentation (dark green) and automatic tumor segmentation (red)

**Fig. 4:**
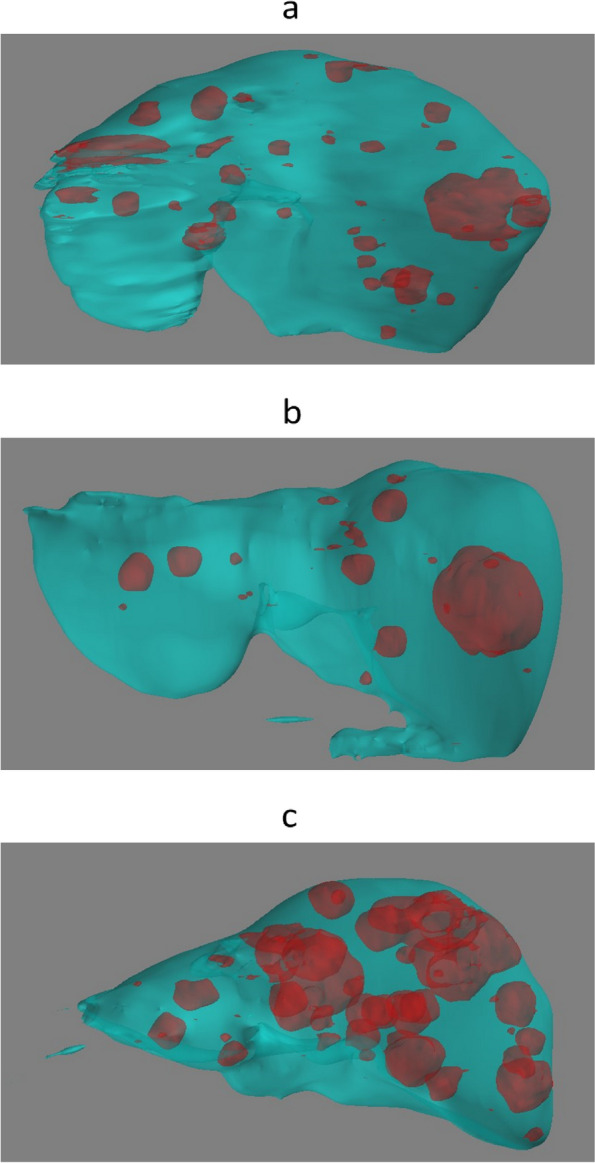
Three-dimensional visualizations of the automatic segmentation of three patients in the development cohort in coronal posterior-anterior view. **a–c** Automatic liver segmentation (blue); automatic tumor segmentation (red)

**Fig. 5:**
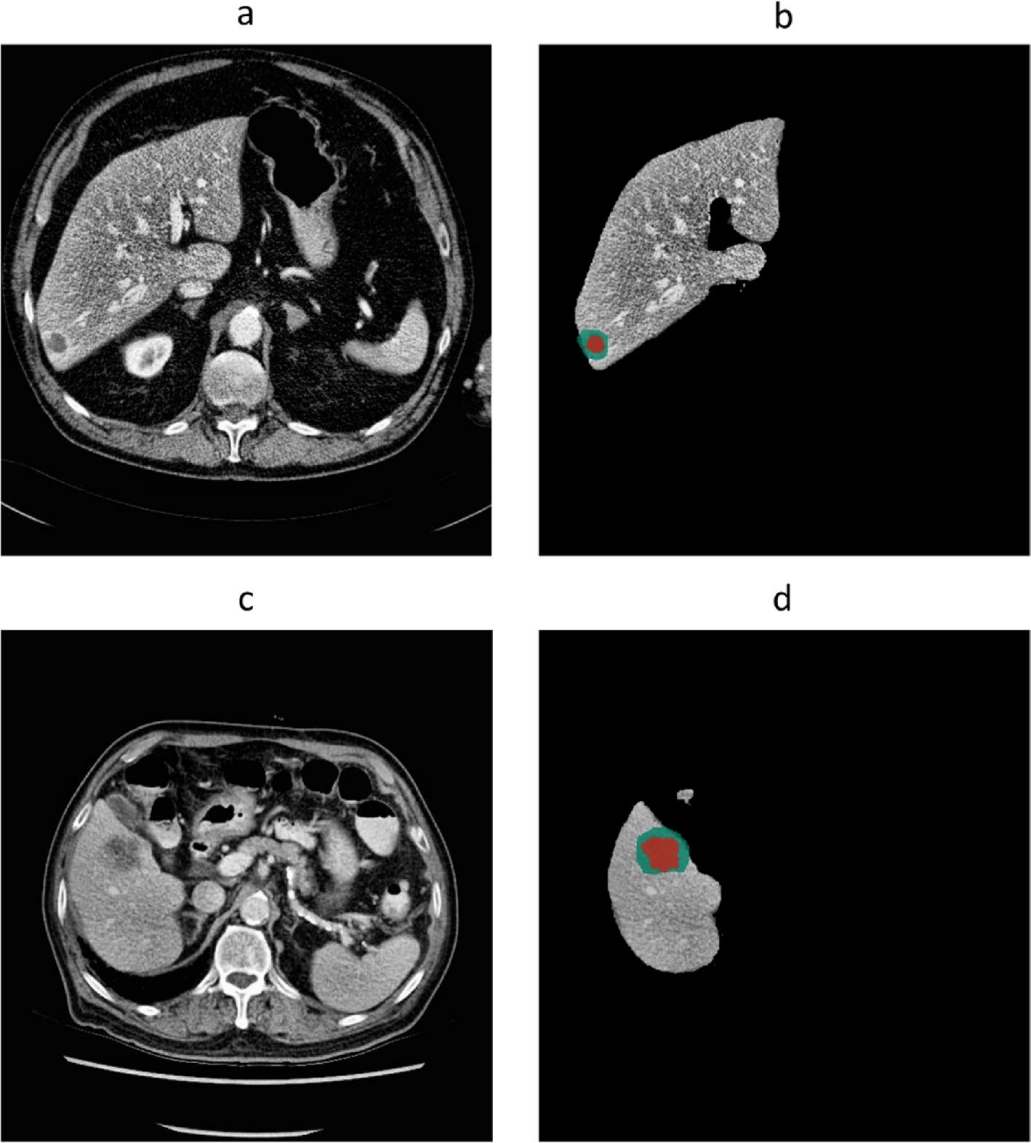
Automatic segmentations in two patients of the external validation cohort. **a**, **c** Computed tomography scans before automatic segmentation. **b**, **d** Radiologist’s tumor segmentation (dark green) and automatic tumor segmentation (red)

In the test set, the spatial agreement assessment of the autosegmentation models had a global DSC of 0.96 and 0.86 for liver and CRLM segmentation, respectively. The corresponding median per-case DSCs were 0.96 (IQR 0.95–0.96) and 0.80 (IQR 0.67–0.87). The intersection-over-union, precision, and recall were 0.75, 0.89, and 0.84 for tumor segmentation, respectively (Table 2). In Fig. 3, examples of the automatic segmentations of the liver and CRLM in the development cohort are depicted. Figure 4 illustrates a 3D visualization of automated liver and CRLM segmentations for three patients. The external validation cohort contained 72 CT scans. The autosegmentation tumor model resulted in a global DSC of 0.82 for CRLM segmentation, with a corresponding median per-case DSC of 0.60 (IQR 0.27–0.76). The intersection-over-union, precision, and recall were 0.69, 0.85, and 0.78 for tumor segmentation, respectively (Table 2). Figure 5 shows examples of the CRLM segmentation in two patients of the external validation.

**Table 2:** Image segmentation evaluation metrics of the tumor model in the development cohort and external validation cohort

	Development cohort (test set)	External validation cohort
Global DSC	0.86	0.82
Per-case DSC (IQR)	0.80 (0.67−0.87)	0.60 (0.29−0.76)
Intersection-over-union	0.75	0.69
Precision	0.89	0.85
Recall	0.84	0.78
True positive (voxels)	13,170,769	733,046
False positive (voxels)	1,755,261	127,677
False negative (voxels)	2,553,727	203,102
True negative (voxels)	96,3282,7315	2,631,648,367

*DSC* Dice similarity coefficient, *IQR* Interquartile range

### Total tumor volume assessment

An excellent agreement was found between reference and automated TTV in the test set of the development cohort (ICC 0.97, confidence interval 95% 0.96–0.98) and in the external validation cohort (ICC 0.98, confidence interval 95% 0.96–0.99). In the development cohort, no significant difference (*p* = 0.632) was found in the reference TTV between the training cohort and the test set (Table 3).

**Table 3:** Total tumor volume assessment

	**Number of CT scans**	**Total tumor volume (cm**^**3**^**)**
Radiologist		
Training cohort	473	67.64 (16.77–302.36)
Test set	122	66.99 (15.84–204.67)
External validation cohort	72	5.65 (2.29–17.19)
Autosegmentation tumor model		
Test set	122	58.49 (14.61–195.97)
External validation cohort	72	7.91 (2.96–20.50)
Difference reference and automatic volume		
Test set	122	7.34 (2.82–21.67)
External validation cohort	72	2.42 (0.80–6.06)

Values are shown as median (interquartile range, 25th−75th percentile). The training cohort consisted of CT scans from the training set and validation set, as both sets were used for model training*. CT* Computed tomography

### Agreement between different observers

An excellent agreement in segmentation was found between the four independent expert abdominal radiologists in 41 scans of 20 patients. The per-case DSC ranged between 0.90 and 0.94 and the global DSC ranged between 0.91 and 0.94. The per-case DSC between the radiologist determining the ground truth and the three independent expert abdominal radiologists was 0.90, 0.92, and 0.91 (Table 4). In addition, a median per-case DSC of 0.99 was observed between the segmentations of the research team and the expert radiologist determining the ground truth.

**Table 4:** Per-case Dice similarity coefficients [IQR]/global) between couples of four independent expert abdominal radiologists

	**Radiologist 1**	**Radiologist 2**	**Radiologist 3**	**Radiologist 4**
Radiologist 1	−	0.90 (0.87−0.93)/0.91	0.92 0.90−0.95)/0.92	0.91 (0.89−0.94)/0.94
Radiologist 2	0.90 (0.87−0.93)/0.91	−	0.94 (0.90−0.96)/0.92	0.93 (0.88−0.96)/0.93
Radiologist 3	0.92 (0.90−0.95)/0.92	0.94 (0.90−0.96)/0.92	−	0.94 (0.90−0.97)/0.93
Radiologist 4	0.91 (0.89−0.94)/0.94	0.93 (0.88−0.96)/0.93	0.94 (0.90−0.97)/0.93	−

Radiologist 1 is the observer determining the ground truth in the development of the model

Radiologists 2, 3, and 4 are the three additional abdominal radiologists. *IQR* Interquartile range (25th−75th percentile)

## Discussion

In this study, deep learning models were successfully developed to segment the liver and CRLM automatically and accurately in CT scans of patients suffering from initially unresectable CRLM. Moreover, the models enabled automatic assessment of TTV of all the CRLM in those CT scans with an excellent agreement with the radiologist’s assessment. In the external validation cohort, consisting of patients with upfront resectable CRLM, the models performed less accurately than in the test set of the development cohort.

The performances of the autosegmentation models in the CAIRO5 test set of this study were comparable or superior to autosegmentation models for liver and liver tumor segmentation in earlier studies [24–26]. In the LiTS, the best liver segmentation model scored a per-case DSC of 0.97, and the best tumor segmentation model scored a per-case DSC of 0.83 [25]. In contrast to this study, the LiTS Benchmark dataset contained imaging data of patients with different types of liver tumors.

The autosegmentation tumor model in the current study obtained lower DSCs in the external validation cohort. This could be explained by the different types of patients in the two data sets. The autosegmentation models were trained and tested on data consisting of pre- and post-treatment CT scans of patients with initially unresectable CRLM [28]. This patient group was initially not suitable for local therapy because of disease extensiveness and the liver CT scans were often complicated by confluent tumors and extensive numbers of CRLM. As a result, patients with a small number of metastases were underrepresented. We hypothesized that the autosegmentation tumor model capable of segmenting patients with extensive CRLM would also be capable of segmenting patients with less extensive disease.

The median smaller size of CRLM included in the external validation cohort could also be a reason for the lower DSCs. This was also demonstrated in the study of Vorontsov et al. [27], who developed deep learning models with the same U-net-architecture for automatic segmentation of CRLM in CT scans. In the test set of their study, the automatic model performed better in lesions larger than 20 mm as compared to lesions smaller than 10 mm or between 10 and 20 mm, obtaining per-lesion DSCs of 0.68, 0.14, and 0.53, respectively.

Autosegmentation remains a challenging task due to variable image parameters, patient variability, and tumor morphology. Therefore, autosegmentation models should be trained on CT scan data that is as realistic and robust as possible. In the current study, the CT acquisition parameters varied considerably across the 54 centers in the development cohort, since scans were performed using different CT scanners and acquisition protocols. However, all scans were of adequate quality to be used for patient management. The variety in CT acquisition parameters is a good representation of CT scans in daily practice and could be considered as a strength with respect to external validity.

The autosegmentation models allowed for the automatic assessment of TTV, not only leading to a more advanced interpretation of change in tumor size, as the effect on all tumorous tissue of all metastases is taken into account. In addition, this method is potentially also less subjective, tedious, and time-consuming than tumor response assessments by radiologists in the future. Assessment of TTV response to systemic therapy could represent a clinically more reliable tumor evaluation than RECIST1.1, as it was shown to be prognostic for recurrence-free survival, whilst RECIST1.1 was not [7]. Moreover, the autosegmentation models can enable the automatic assessment of other relevant imaging features for tumor response evaluation, such as morphological changes [5, 37]. Besides improving tumor response evaluation, the autosegmentation models could also play a role in radiomics research. Tumor segmentation forms an important step in the process of radiomics, in which hundreds of imaging features can be analyzed out of tumor segmentations and used in predictive modeling through machine learning [38–40].

It is important to emphasize that the autosegmentation models in the current study have been developed to improve tumor response evaluation of CRLM and not to diagnose CRLM. Models capable of diagnosing CRLM require a different approach with an extensive amount of data comprising different benign and malignant types of liver lesions.

During the design of the current study, the U-net was the state-of-the-art architecture, and a 2D U-net was employed instead of a 3D U-net. Recently, other architectures like the U-net +  + and Trans U-net were developed, so it could be considered to make use of such architectures in the future. Moreover, the 2D U-Net was preferred over the 3D U-Net as it is more accurate specifically for the liver and requires less computational power [41].

The present study has several limitations. Firstly, the ground truth was based on the evaluation and adjustment of one expert radiologist. Consequently, the ground truth of one observer had a large influence on model training and ultimately model performance. The original study of the external CRLM cohort already reported significant differences between the segmentations of different observers [30]. However, excellent agreement in tumor segmentation was observed between four independent expert radiologists and it was not logistically feasible to base the ground truth on the segmentations of multiple radiologists. Therefore, it was chosen to determine the ground truth based on one radiologist. Secondly, a selection of patients with initially unresectable CRLM was used for model training. This may have influenced the generalizability of the developed autosegmentation tumor model, as it performed less in the external cohort consisting of patients with resectable and fewer number of CRLM. However, the autosegmentation models are developed to improve the evaluation of CRLM to systemic therapy. Patients with CRLM receiving systemic treatment often have more extensive disease or large tumors. Finally, to enhance density differences between the liver and tumors we have applied histogram equalization. However, this approach may have reduced the (calibrated) intensity values in the images for the segmentation steps. With the high accuracies obtained in our study, we do not expect that this pre-processing step has negatively influenced the segmentation agreement.

In the future, the actual implementation of an automatic tumor response pipeline into clinical care will face challenges such as technical feasibility, ethical concerns, and regulatory aspects [42, 43]. A potential first step to implementation is to conduct a prospective clinical study with an integrated tumor response pipeline with a human-in-the-loop situation. Moreover, if the automatic tumor response pipeline is implemented successfully and has proven to be clinically relevant, the autosegmentation model could be translated to other imaging modalities (*e.g.*, MRI).

In conclusion, the deep learning models developed in this study were able to automatically segment the liver and CRLM with high accuracy in patients with initially unresectable CRLM. This has a high potential of decreasing radiologist’s workload and increasing accuracy by lowering interobserver variability. Moreover, the models enabled automatic assessment of TTV and the response of TTV to systemic treatment. This and other potentially highly relevant imaging features, such as tumor morphological response could potentially contribute to more consistent and clinically relevant tumor response assessments for patients with CRLM receiving systemic treatment in future clinical care and research.

### Supplementary Information

**Additional file 1:S1.** Inclusion and exclusion criteria of the CAIRO5 trial^[1]^. **S2.** CT parameters. **S3.** Model details. **S4.** Scans in training, validation and test set. **S5.** Flow diagram patient selection external validation cohort.

## Data Availability

The datasets used and/or analyzed during the current study are available from the corresponding author on reasonable request.

## Abbreviations

CRLM: Colorectal liver metastases
CT: Computed tomography
DSC: Dice similarity coefficient
ICC: Intraclass correlation coefficient
IQR: Interquartile range
LiTS: Liver Tumor Segmentation Challenge
MRI: Magnetic resonance imaging
TTV: Total tumor volume
