# Supplementary material for: Deep learning models for automatic tumor segmentation and total tumor volume assessment in patients with colorectal liver metastases
Source: Eur Radiol Exp. 2023 Dec 1;7:75. doi: 10.1186/s41747-023-00383-4 (PMC10692044; doi:10.1186/s41747-023-00383-4)
Supplement: Supplementary file 1 — Additional file 1:S1. Inclusion and exclusion criteria of the CAIRO5 trial[1]. S2. CT parameters. S3. Model details. S4. Scans in training, validation and test set. S5. Flow diagram patient selection external validation cohort. [file 41747_2023_383_MOESM1_ESM.docx]

**SUPPLEMENT (S1-S5)**

**S1. Inclusion and exclusion criteria of the CAIRO5 trial^[1]^**Inclusion criteria: histological proof of colorectal cancer, previously untreated and unresectable metastases confined to the liver (as assessed by the central panel) according to CT scan obtained less than 2 weeks prior to registration, adequate tumour tissue available for assessment of *RAS* and *BRAF* mutation status, WHO performance status 0-1 (Karnofsky performance status ≥ 70), age ≥ 18 years, no contraindications for liver surgery, resectable primary tumour if still *in situ*, adequate organ functions, life expectancy over 12 weeks, expected adequacy of follow-up, and written informed consent.

Exclusion criteria: previous systemic treatment for metastatic disease, extrahepatic metastases, with the exception of small (≤1 cm) extrahepatic lesions that are not suspicious of metastases, unresectable primary tumour, serious comorbidity or any other condition preventing the safe administration of study treatment (including both systemic treatment and surgery), major cardiovascular event within 12 months before randomisation, uncontrolled hypertension, or unsatisfactory blood pressure control with ≥3 antihypertensive drugs, previous adjuvant treatment unless completed ≥ 6 months prior to randomisation, previous surgery for metastatic disease, previous intolerance of study drugs in the adjuvant setting, pregnant or lactating women, second primary malignancy within the past 5 years with the exception of adequately treated in situ carcinoma of any organ or basal cell carcinoma of the skin, any concomitant experimental treatment.

1. Huiskens J, van Gulik TM, van Lienden KP, Engelbrecht MR, Meijer GA, van Grieken NC, et al. Treatment strategies in colorectal cancer patients with initially unresectable liver-only metastases, a study protocol of the randomised phase 3 CAIRO5 study of the Dutch Colorectal Cancer Group (DCCG). BMC Cancer. 2015;15:365. doi: 10.1186/s12885-015-1323-9.

**S2. CT parameters**

| **Development cohort (CAIRO5)** |  |
| --- | --- |
| Slice thickness (mm) | 5.0 [3.0 – 5.0] |
| Pixel spacing (mm) | 0.73 [0.68 – 0.78] |
| Tube voltage (kvP) | 120 [100 – 120] |
| Total collimation width (mm) | 40 [40 – 40] |
| Exposure (mAs) | 50 – 293 |
| CT dose index (mGy) | 2 – 15 |

Note – Values are displayed as median with [interquartile range] or as range.

| **External validation cohort** |  |
| --- | --- |
| Slice thickness (mm) | 3.0 [3.0 – 5.0] |
| Pixel spacing (mm) | 0.74 [0.68 – 0.78] |
| Tube voltage (kvP) | 120 [120 – 120] |
| Tube current (mA) | 239 [143 – 325] |

Note – Values are displayed as median with [interquartile range].

**S3. Model details**

| **Model** |  |
| --- | --- |
| Architecture | U-net ^[2]^ |
| Input resolution | 512x512 |
| Output resolution | 512x512 |
| Number of convolutional layers | 23 |
|  |  |
| **Solver** |  |
| Solver type | Adam |
| Loss function | Entropy |
| Learning rate | 0.0001 |
| Minimum gradient value | -100 |
| Maximum gradient value | 100 |
|  |  |
| **Optimizer** |  |
| L1 | 0 |
| L2 | 0.0001 |
| Mini batch size | 1 |
| Dropout | 0 |

2. Ronneberger O, Fischer P, Brox T. U-Net: Convolutional Networks for Biomedical Image Segmentation. In: Navab N, Hornegger J, Wells WM, Frangi AF, editors. Medical Image Computing and Computer-Assisted Intervention – MICCAI 2015. Cham: Springer International Publishing; 2015. p. 234-41.

**S4. Scans in training, validation and test set**

| **Patient** | **Number of training scans** | **Number of validation scans** | **Number of test scans** |
| --- | --- | --- | --- |
| **001** | 2 |  |  |
| **004** | 2 |  |  |
| **007** | 2 |  |  |
| **010** | 2 |  |  |
| **011** | 3 |  |  |
| **012** | 2 |  |  |
| **014** | 2 |  |  |
| **015** | 2 |  |  |
| **017** | 2 |  |  |
| **018** | 3 |  |  |
| **021** | 2 |  |  |
| **022** | 3 |  |  |
| **026** | 2 |  |  |
| **027** | 2 |  |  |
| **028** | 2 |  |  |
| **029** | 2 |  |  |
| **030** | 2 |  |  |
| **032** | 2 |  |  |
| **033** | 3 |  |  |
| **034** | 2 |  |  |
| **036** | 2 |  |  |
| **037** | 2 |  |  |
| **038** | 2 |  |  |
| **041** | 2 |  |  |
| **043** | 2 |  |  |
| **044** | 2 |  |  |
| **045** | 2 |  |  |
| **046** |  |  | 2 |
| **047** |  |  | 3 |
| **048** |  |  | 2 |
| **049** |  |  | 2 |
| **050** | 2 |  |  |
| **051** | 2 |  |  |
| **052** | 1 |  |  |
| **053** | 2 |  |  |
| **054** | 2 |  |  |
| **055** | 2 |  |  |
| **057** | 2 |  |  |
| **058** | 2 |  |  |
| **061** | 1 |  |  |
| **062** | 2 |  |  |
| **063** | 2 |  |  |
| **065** | 3 |  |  |
| **066** | 2 |  |  |
| **067** | 2 |  |  |
| **068** | 2 |  |  |
| **070** | 3 |  |  |
| **072** | 2 |  |  |
| **074** | 2 |  |  |
| **075** | 3 |  |  |
| **076** | 2 |  |  |
| **077** |  |  | 3 |
| **078** |  |  | 3 |
| **079** |  |  | 2 |
| **082** | 2 |  |  |
| **083** | 3 |  |  |
| **084** | 1 | 1 |  |
| **085** | 2 |  |  |
| **086** | 2 |  |  |
| **087** | 3 |  |  |
| **088** | 2 |  |  |
| **089** | 2 |  |  |
| **092** | 2 |  |  |
| **093** | 2 |  |  |
| **095** | 2 |  |  |
| **096** | 1 |  |  |
| **097** | 2 |  |  |
| **101** | 2 |  |  |
| **102** | 3 |  |  |
| **105** | 2 |  |  |
| **107** | 3 |  |  |
| **111** | 3 |  |  |
| **112** | 2 |  |  |
| **116** | 2 |  |  |
| **117** | 2 |  |  |
| **118** | 4 |  |  |
| **121** | 2 |  |  |
| **122** | 3 |  |  |
| **123** | 2 |  |  |
| **124** | 2 |  |  |
| **125** |  |  | 2 |
| **126** |  |  | 2 |
| **127** |  |  | 2 |
| **128** |  |  | 2 |
| **129** |  |  | 2 |
| **130** | 1 | 1 |  |
| **131** |  |  | 4 |
| **132** |  |  | 2 |
| **133** |  |  | 3 |
| **134** |  |  | 2 |
| **135** | 2 |  |  |
| **136** |  | 2 |  |
| **137** |  | 2 |  |
| **138** |  | 2 |  |
| **139** | 2 |  |  |
| **140** | 2 |  |  |
| **141** | 2 |  |  |
| **143** |  | 2 |  |
| **144** | 2 |  |  |
| **148** |  | 2 |  |
| **151** | 2 |  |  |
| **152** | 4 |  |  |
| **153** | 2 |  |  |
| **154** | 4 |  |  |
| **155** | 2 |  |  |
| **157** | 3 |  |  |
| **158** | 2 |  |  |
| **159** | 2 |  |  |
| **161** | 2 |  |  |
| **162** | 2 |  |  |
| **165** | 3 |  |  |
| **166** | 3 |  |  |
| **167** | 2 |  |  |
| **168** | 2 |  |  |
| **169** | 2 |  |  |
| **174** | 2 |  |  |
| **175** | 2 |  |  |
| **176** | 2 |  |  |
| **177** | 2 |  |  |
| **178** | 3 |  |  |
| **180** | 2 |  |  |
| **182** | 2 |  |  |
| **185** | 3 |  |  |
| **186** | 3 |  |  |
| **188** | 2 |  |  |
| **189** | 2 |  |  |
| **191** | 2 |  |  |
| **194** | 3 |  |  |
| **195** | 2 |  |  |
| **197** | 2 |  |  |
| **198** | 2 |  |  |
| **200** | 3 |  |  |
| **202** | 2 |  |  |
| **204** | 2 |  |  |
| **205** | 2 |  |  |
| **207** | 2 |  |  |
| **208** | 3 |  |  |
| **210** | 2 |  |  |
| **211** | 3 |  |  |
| **215** | 2 |  |  |
| **216** |  |  | 2 |
| **218** | 3 |  |  |
| **220** |  |  | 2 |
| **221** |  |  | 2 |
| **222** |  |  | 3 |
| **223** |  |  | 2 |
| **224** |  |  | 2 |
| **225** |  |  | 2 |
| **226** | 4 |  |  |
| **227** |  |  | 2 |
| **229** |  |  | 2 |
| **230** | 2 |  |  |
| **231** |  |  | 2 |
| **232** |  |  | 4 |
| **235** | 1 | 2 |  |
| **236** |  | 3 |  |
| **237** |  | 2 |  |
| **240** | 2 |  |  |
| **241** | 2 |  |  |
| **242** | 4 |  |  |
| **245** | 2 |  |  |
| **247** | 3 |  |  |
| **248** | 2 |  |  |
| **252** | 2 |  |  |
| **253** | 3 |  |  |
| **255** | 3 |  |  |
| **256** | 2 |  |  |
| **257** | 3 |  |  |
| **259** | 2 |  |  |
| **261** | 2 |  |  |
| **262** | 3 |  |  |
| **265** | 2 |  |  |
| **266** | 2 |  |  |
| **267** | 3 |  |  |
| **269** | 3 |  |  |
| **270** | 3 |  |  |
| **272** | 2 |  |  |
| **273** | 2 |  |  |
| **274** | 2 |  |  |
| **277** | 1 |  |  |
| **282** | 2 |  |  |
| **283** | 2 |  |  |
| **284** | 2 |  |  |
| **286** | 2 |  |  |
| **287** |  |  | 2 |
| **291** | 3 |  |  |
| **292** | 2 |  |  |
| **293** | 2 |  |  |
| **295** | 2 |  |  |
| **296** | 2 |  |  |
| **297** |  |  | 3 |
| **298** | 2 |  |  |
| **299** | 4 |  |  |
| **300** | 3 |  |  |
| **302** |  |  | 2 |
| **303** |  |  | 2 |
| **304** | 2 |  |  |
| **305** | 2 |  |  |
| **306** |  |  | 4 |
| **307** |  |  | 2 |
| **308** | 3 |  |  |
| **309** | 2 |  |  |
| **310** | 3 |  |  |
| **311** | 2 |  |  |
| **312** | 3 |  |  |
| **314** | 2 |  |  |
| **315** | 2 |  |  |
| **318** | 3 |  |  |
| **323** | 2 |  |  |
| **324** | 4 |  |  |
| **325** | 4 |  |  |
| **326** | 2 |  |  |
| **327** | 2 |  |  |
| **330** | 2 |  |  |
| **331** |  |  | 2 |
| **332** |  |  | 4 |
| **334** | 2 |  |  |
| **335** |  |  | 2 |
| **336** |  |  | 2 |
| **337** |  |  | 2 |
| **338** |  |  | 2 |
| **339** |  |  | 2 |
| **340** |  |  | 2 |
| **342** |  |  | 2 |
| **344** |  |  | 3 |
| **346** |  |  | 2 |
| **347** |  |  | 2 |
| **348** |  |  | 3 |
| **349** |  |  | 3 |
| **350** |  | 2 |  |
| **351** |  | 4 |  |
| **352** |  | 2 |  |
| **353** | 2 |  |  |
| **354** | 2 |  |  |
| **355** | 2 |  |  |
| **358** | 3 |  |  |
| **359** | 2 |  |  |
| **362** | 3 |  |  |
| **364** | 2 |  |  |
| **365** | 2 |  |  |
| **367** | 3 |  |  |
| **368** | 2 |  |  |
| **369** | 2 |  |  |
| **370** |  |  | 3 |
| **372** | 3 |  |  |
| **374** | 2 |  |  |
| **375** | 2 |  |  |
| **378** | 2 |  |  |
| **379** | 2 |  |  |
| **383** |  | 2 |  |
| **384** |  | 3 |  |
| **386** | 1 | 1 |  |
| **388** |  | 2 |  |
| **389** |  |  | 2 |
| **390** |  |  | 2 |
| **396** |  |  | 2 |
| **400** |  |  | 2 |
| **406** |  | 2 |  |
| **407** |  | 2 |  |

**
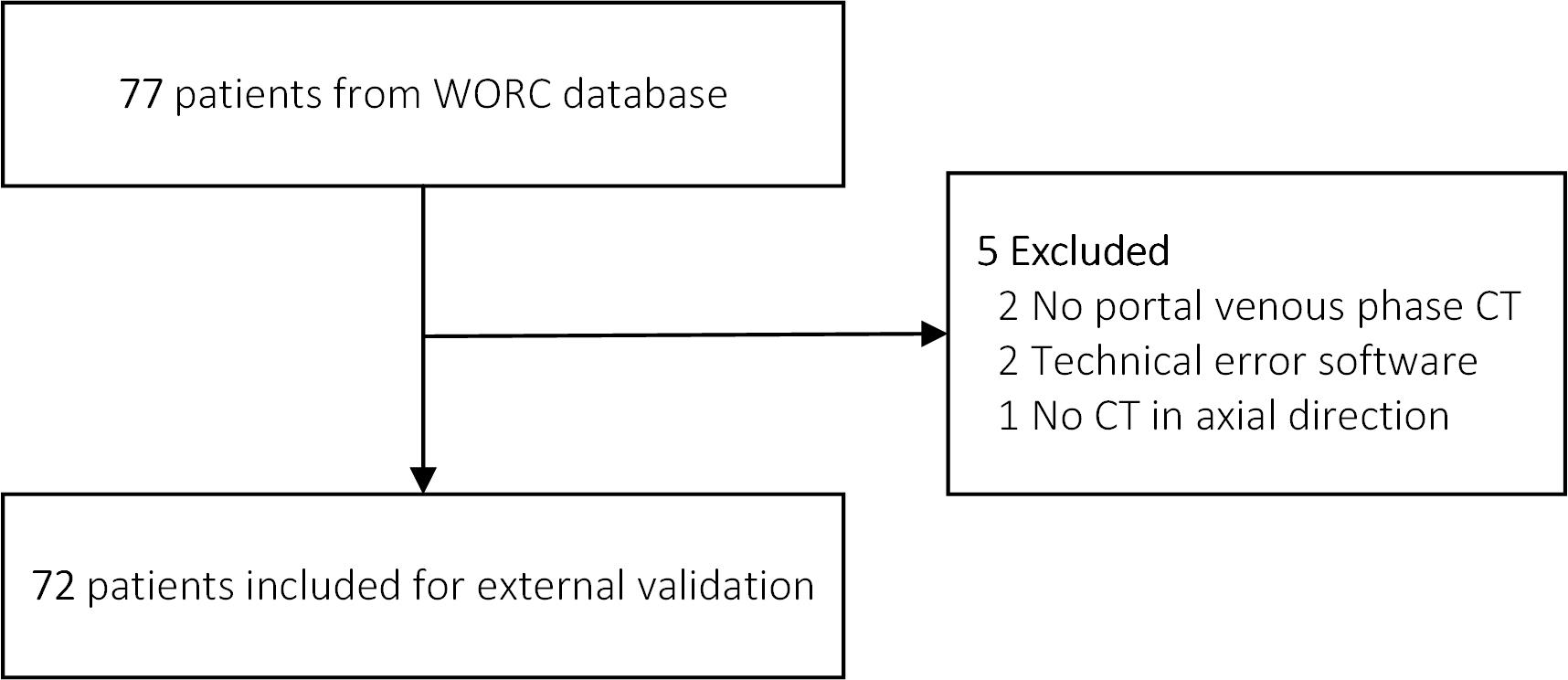
S5. Flow diagram patient selection external validation cohort**

Abbreviations: CT, computed tomography; WORC, Workflow for Optimal Radiomics Classification.
